# Comparative Analysis of the Hematopoietic Progenitor Cells from Placenta, Cord Blood, and Fetal Liver, Based on Their Immunophenotype

**DOI:** 10.1155/2015/418752

**Published:** 2015-08-05

**Authors:** Maria D. Kuchma, Vitaliy M. Kyryk, Hanna M. Svitina, Yulia M. Shablii, Lubov L. Lukash, Galina S. Lobyntseva, Volodymyr A. Shablii

**Affiliations:** ^1^Institute of Cell Therapy, Komarova Avenue 3, Kyiv 03680, Ukraine; ^2^Institute of Molecular Biology and Genetics, National Academy of Sciences of Ukraine, Zabolotnogo Street 150, Kyiv 03680, Ukraine; ^3^State Institute of Genetics and Regenerative Medicine, National Academy of Medical Sciences of Ukraine, Vyshgorodska Street 67, Kyiv 04114, Ukraine

## Abstract

We have investigated the characteristics of human hematopoietic progenitor cells (HPCs) with the CD34^+^CD45^low^SSC^low^ phenotype from full-term placental tissue (FTPT) as compared to cord blood (CB) and fetal liver (FL) cells. We demonstrated the presence of cell subpopulations at various stages of the differentiation with such immunophenotypes as CD34^+/low^CD45^low/−^, CD34^++^CD45^low/−^, CD34^+++^CD45^low/−^, CD34^+/low^CD45^hi^, and CD34^++^CD45^hi^ in both first trimester placental tissue (FiTPT) and FTPT which implies their higher phenotypic heterogeneity compared to CB. HPCs of the FTPT origin expressed the CD90 antigen at a higher level compared to its expression by the CB HPCs and the CD133 antigen expression being at the same level in both cases. The HPCs compartment of FTPT versus CB contained higher number of myeloid and erythroid committed cells but lower number of myeloid and lymphoid ones compared to FL HPCs. HPCs of the FTPT and CB origin possess similar potentials for the multilineage differentiation *in vitro* and similar ratios of myeloid and erythroid progenitors among the committed cells. This observation suggests that the active hematopoiesis occurs in the FTPT. We obtained viable HPCs from cryopreserved placental tissue fragments allowing us to develop procedures for banking and testing of placenta-derived HPCs for clinical use.

## 1. Introduction

Deficiency of donors of population of hematopoietic progenitor cells that contain stem ones (HPCs) needed for transplantation in cases of oncohematological diseases and congenital hematologic disorders remains one of the most important problems in hematology. Although HPCs of bone marrow origin are widely used for transplantations, limitations in HLA-identical bone marrow grafts still pose a big challenge. HPCs of mobilized peripheral blood from patients who were treated using chemotherapy and/or cytokines administration are also used [1]. However, a very critical moment of this process is the frame term of from 3 to 6 months from the beginning of the HPCs samples search (i.e., of bone marrow and mobilized peripheral blood) up to transplantation and at the same time obtaining the HPCs has risks for donors [2]. Since 1988, cord blood (CB) has become a source of HPCs and nowadays it is widely used for transplantations [3]. Advantages of this source include the easiness and safety of CB sample obtaining, the possibility for immediate use of stored HLA-typed units in CB banks [3], lower requirements for HLA matching, and the lower incidence of graft-versus-host disease [2, 3]. However, there are some disadvantages accompanying the CB cells transplantation which include limited quantities of collected HPCs, delayed engrafting of neutrophils, platelets, and immune cells, as well as higher rate of graft failure [3]. It has been reported that the fetal liver (FL) as a rich source of HPCs [4, 5] can give encouraging results following transplantation to humans both before or after birth with immunodeficiency disease, with severe aplastic anemia, or with inborn errors of metabolism [6, 7]; but there is no convicting data concerning the human FL HPCs engrafting in adult niche such as bone marrow. In addition, the FL HPC transplantation is problematic because of ethical considerations; therefore the procedure for obtaining these cells is a sophisticated one and their quantities are small [5]. Therefore, the search for new additional HPC sources is important for medicine. Human placenta has become known to play an important role in fetal hematopoiesis [8, 9] and is considered to be used as a potential additional source of HPCs for transplantation [10]. To evaluate the possibility of FTPT HPCs application for clinical purposes, it is necessary to investigate their properties and characteristics and it is important to compare their properties with those of fetal HPCs, especially of hematopoietic cells that are currently used for transplantation. It is also necessary to develop methods for their preservation for further application.

Therefore, the aim of our study was the comparative analysis of HPCs from FTPT, first-trimester placental tissue (FiTPT), CB, FL, and characterization of HPCs from cryopreserved placental tissue.

## 2. Materials and Methods

### 2.1. Obtaining of Cell Fraction from FTPT, FiTPT, and CB

The Committee of Human Research of the Institute of Cell Therapy has approved this study and consent procedure (#3-13). The placentas (*n* = 16) and CB were received from the Kyiv City Maternity Hospital #3 after full-term deliveries (physiological or by cesarean section) from 23–36 years old women at 39–41 weeks of gestation upon their written informed consent. The CB samples (*n* = 15) were collected by the standard methods of CB sampling. The first-trimester placentas (*n* = 3) were obtained from elective aborted human embryos at 6 to 12 weeks of gestation upon the women's written informed consent (City Clinical Hospital #2, Kyiv).

Results of anti-HIV-1/2, HIV-1/2 RNA, anti-HCV, HCV RNA, anti-HBcor, HBsAg, and anti-*Treponema pallidum* assays were negative for women whose samples of the CB and the placentas were used in this research.* Chlamydia trachomatis*,* Mycoplasma genitalium*,* Ureaplasma *sp., and* HSV-1/2* were not found by the NAT method in their FTPT.

The umbilical cord was cut off and removed from the FTPT together with amnion. To remove the blood, the remaining FTPT was minced by sterile scissors into small fragments (1–3 mm) and washed intensively on a shaker by Hanks' balanced salt solution (HBSS) (HyClone, USA) supplemented with penicillin (100 U/mL) and streptomycin (50 mg/mL) until the washing solution became colorless.

For native FTPT and FiTPT and cryopreserved FTPT samples the tissue was put into phosphate buffered saline (PBS) (HyClone, USA) with the addition of 0.2% collagenase I (Serva, Germany), 0.35 mg/mL hyaluronidase (Sigma, USA), 100 U/mL DNase I (Sigma, USA), and 1 mg/mL bovine serum albumin (BSA) (Carl Roth, Germany) and were treated for 30–50 minutes at +37°C (method #1). The placental cells were then isolated by filtration through a 70 *μ*m nylon cell strainer (Becton Dickinson, USA). At the 2nd stage, the remaining tissue was incubated with a fresh portion of enzymes for 20–40 minutes at +37°C. The enzyme activity was inhibited by the addition of fetal bovine serum (FBS) (HyClone, USA) and the cells were washed in BSA-containing (1 mg/mL) PBS by centrifugation at 400 g for 5 min. The cell fractions obtained after both stages of the enzymatic treatment were then pooled.

The native FTPT was treated in the Dulbecco's Modified Eagle Medium (DMEM) (HyClone, USA) with the addition of 5 mM HEPES (Biomedicals, France), 0.1% collagenase I, and 0.6 unit/mL dispase I (Gibco, Germany) for 10–30 minutes at +37°C (method #2). The enzyme activity was inhibited by addition of FBS. Cells were passed through a 70 *μ*m nylon cell strainer and washed in DMEM supplemented with 5 mM HEPES by centrifugation at 400 g for 5 min.

The suspension of FTPT cells after treatment by method #1, 2 and CB were fractionated with Ficoll density gradient (1.077 g/mL, Biochrome, Germany) at 400 g for 30 min. The light-density cell fraction was washed twice in the PBS supplemented with BSA (1 mg/mL).

The frozen-thawed FTPT and FiTPT were treated in the DMEM with the addition of 0.1% collagenase I and 5 mM HEPES for 7–10 minutes at +37°C. The enzyme activity was inhibited by addition of FBS. The cells were isolated by filtration through a 70 *μ*m cell strainer and washed in DMEM supplemented with 5 mM HEPES by centrifugation at 300 g for 5 min. The pellet was suspended in the DMEM with HEPES (method #3).

The light-density cell fraction of CB origin was treated with the same enzyme mixture according to the method #1 during 50 min at +37°C to eliminate any potential difference in the FACS result due to enzymatic effect on placental cells. Thereafter the CB light-density cell fraction was centrifuged at 300 g for 10 min and the pellet was resuspended in the BSA-containing (1 mg/mL) PBS.

### 2.2. The FTPT and the FiTPT Cryopreservation and Thawing

For cryopreservation, a HBSS contained 10% DMSO (Sigma, USA) was added slowly to a HBSS with small washed FiTPT or FTPT fragments prepared as described above to the final DMSO concentrations of 5%. The samples were frozen in cryogenic vials following a special program in a software-based freezer (IceCube, Australia). When the temperature reached −140°C, the cooling process in the freezer was stopped and the samples were transferred to long-term storage in liquid nitrogen (−196°C). Thawing was performed in a water bath (+38–40°C) until the liquid phase (0°C) was reached and the DMSO was then removed by slowly adding HBSS to the tissue.

### 2.3. Obtaining the Cells from the FL

Human FL samples (*n* = 7) were collected from elective aborted embryos at 6–12 weeks of gestation upon the women's written informed consent (City Clinical Hospital #2, Kyiv). The FL cells were obtained by FL tissue homogenization in a Potter homogenizer in the HBSS followed by filtration through a 100 *μ*m cell strainer (BD, USA). HIV-1, HCV, HBV, CMV, HSV-1/2, EBV,* Treponema pallidum*,* Toxoplasma gondii*,* Chlamydia trachomatis*,* Mycoplasma genitalium*, and* Ureaplasma *sp. assays results of FL samples were negative. The cryopreservation procedure for liver cells was conducted according to a previously developed three-stage program [11]. The samples were stored in liquid nitrogen (−196°C). The FL cells were thawed in a water bath as described above for the FTPT.

### 2.4. Colony-Forming Cell Assays

The light-density cell fractions from tissue samples of native FTPT (method #1), CB, and the suspension of enzymatically isolated cells from frozen-thawed FTPT and FiTPT (method #3) were cultured in Petri dishes (35 mm, 1.25–5.00 · 10^4^ cells/dish) in the methylcellulose medium MethoCult (Stem Cell Technologies, Canada) at +37°С in the atmosphere of 5%  СО_2_. All types of morphologically identified colonies (BFU-E, CFU-E, CFU-GM, CFU-G, CFU-M, CFU-GEMM) were scored with an inverted microscope after 14 days of culturing.

### 2.5. Flow Cytometry Analysis

The method #1 and #2 were used for obtaining cells from native and cryopreserved placental samples for flow cytometry. For immunophenotyping of cells from tissues studied (FTPT, FiTPT, CB, and FL), we used the following fluorochrome-labeled monoclonal antibodies (Becton Dickinson, USA): anti-CD34 APC, anti-CD90 FITC, anti-CD45 APC-Cy7, anti-CD14 Pacific Blue, anti-CD31 PE, anti-CD133 PE, anti-CD45RA FITC, anti-CD7 PE, anti-CD19 PE-Cy7, anti-CD33 FITC, and anti-CD235a PE. The analysis was performed only for cell populations excluding 7-AAD dye. Immunophenotyping was performed on a cell sorter BD FACSAria (Becton Dickinson, USA) using the BD FACS Diva 6.1.2 software and analyzing simultaneously 2 parameters of light scattering and 7 parameters of fluorescence. Control samples were used to adjust the compensation of fluorochrome overlap: unstained, single stained, fluorescence minus one, and isotype controls. “Expression level” means the percentage of cells expressing a particular antigen. “Intensity of expression” represents the intensity of expression of a particular antigen by a specific cell population.

### 2.6. FISH Analysis

XY FISH analysis was performed on cells isolated from colonies arisen from male light-density FTPT cells in the MethoCult medium after 14 days of growth. Hybridization was performed with human specific centromeric probe CEPХ SpectrumOrange probe and CEPY SpectrumGreen probe (Abbot Molecular, USA) according to the manufacturer's protocol. Nuclei were visualized by DAPI (Abbot Molecular, USA) staining. Signals were visualized using a fluorescence microscope Zeiss Axio Imager.M1 (Carl Zeiss, USA). A total of 300 cells per two placenta samples were examined.

### 2.7. Statistical Analysis

The results were presented as mean values with 95%-confidence interval. The statistically significant differences between the groups were determined using the nonparametric statistics (Mann–Whitney *U* test). The differences at *p* < 0.05 were considered as statistically significant. Statistical analyses were performed with STATISTICA 8.0 software (StatSoft Inc. 2007, USA).

## 3. Results

In our investigation we chose the single-platform ISHAGE (International Society of Hematotherapy and Graft Engineering) protocol for analyzing the immunophenotypes of the placental HPCs as well as those of the CB and FL origin. The population of alive placental CD45^+^ cells contained morphologically different cell subpopulations whose FSC (forward scatter) and SSC (side scatter) characteristics were similar to characteristics of granulocyte, lymphocyte, and monocyte cells as we have previously demonstrated [12].

**Figure 1:**
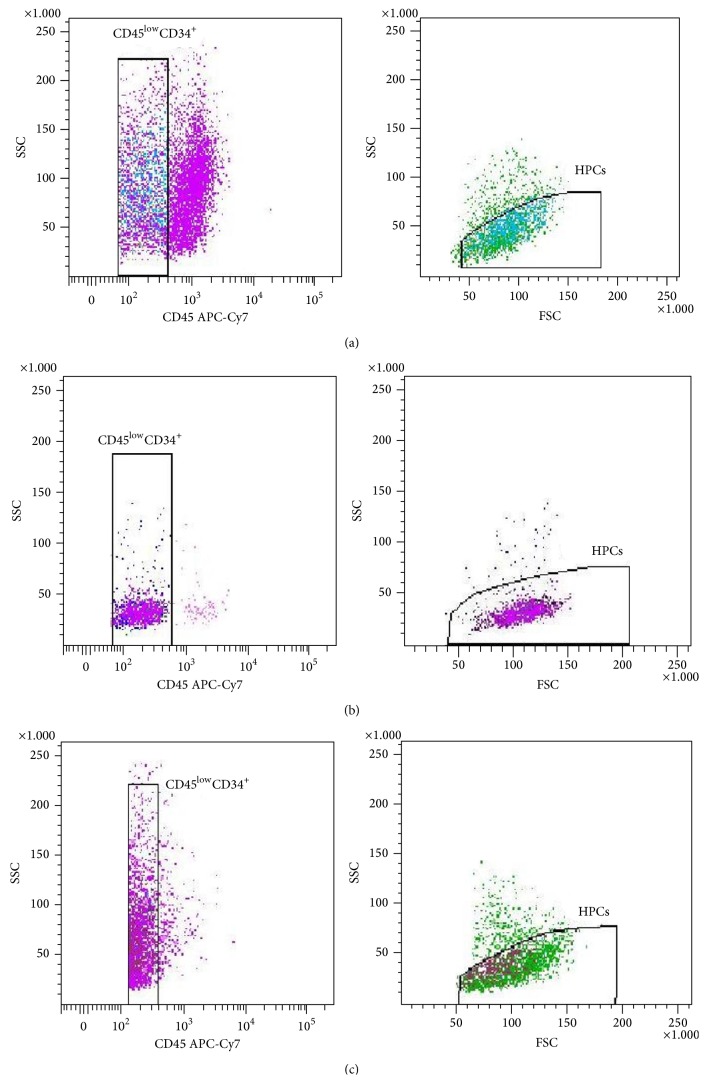
FACS analysis shows that the HPCs subset among the CD34^+^CD45^low^ cell population is the same in the FTPT (a) and the FL (c), but significantly lower compared to the CB (b).

In this study we have shown that according to the ISHAGE protocol the HPCs subset (CD34^+^CD45^low^SSC^low^) among all viable CD45^+^ cells isolated by methods #1 and #2 from the FTPT reaches 0.56% (0.39–0.76%, *n* = 16) and 1.14% (0.29–2.55%, *n* = 5), respectively (there is no statistically significant difference). The content of the HPCs among CD34^+^CD45^low^ cell population was 79.7% (73.4–85.3%, *n* = 16) and 78.9% (58.6–93.6%, *n* = 6) for FTPT and FL, respectively, that was almost the same, but significantly lower compared to the CB (*p* < 0.05) 94.7% (90.9–97.6%, *n* = 15) (Figure 1).

The percentage of total CD34^+^ cells in FTPT (including both CD45^−^ and CD45^+^ cells) reach 1.54% (1.15–1.98%, *n* = 11). We suggested such high percentage can be explained by presence of endothelial cells in this population that also express CD34^+^ [13].

**Figure 2:**
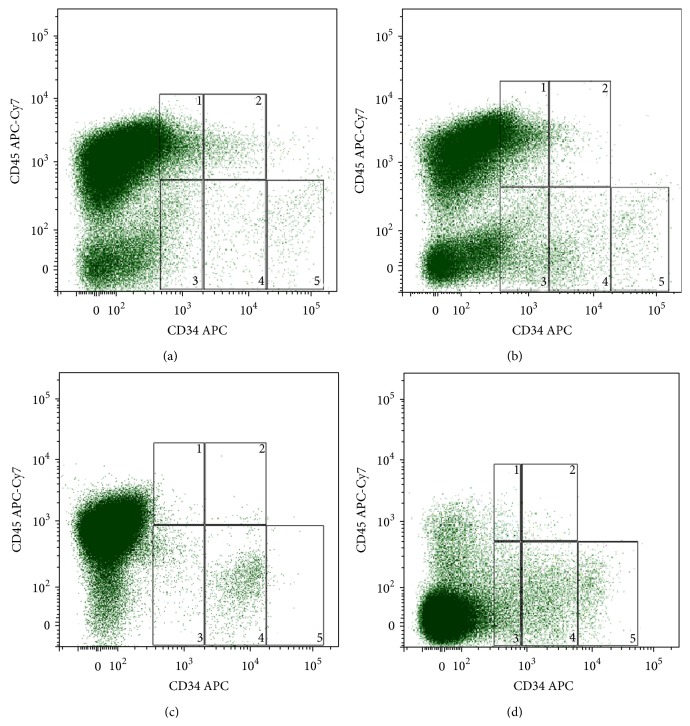
The FTPT (a) and FiTPT (b) contained CD34^+/low^CD45^hi^ (1) and CD34^++^CD45^hi^ (2) cells. In addition, CD34^+/low^CD45^low/−^ (3), CD34^++^CD45^low/−^ (4), and CD34^+++^CD45^low/−^ (5) cell populations were common for FTPT (a), FiTPT (b), and FL (d). CB contained CD34^+/low^CD45^low/−^ (3) and CD34^++^CD45^low/−^ (4) cells but did not contain CD34^+++^CD45^low/−^ (5) cells (c).

We have previously first demonstrated the FTPT to contain three subpopulations that differ by intensity of CD34 expression and could be identified as CD34^+/low^CD45^low/−^, CD34^++^CD45^low/−^, and CD34^+++^CD45^low/−^ (Figure 2(a)) [14]. In this work we have found that the FiTPT also contains these subpopulations (Figure 2(b)). Two subpopulations, namely, CD34^+/low^CD45^low/−^ and CD34^++^CD45^low/−^, have also been detected in the CB and the FL (Figures 2(c) and 2(d)), but the cell subpopulation with the CD34^+++^CD45^low/−^ phenotype being absent in CB (Figure 2(c)). We assume that the FL contains also CD34^+/low^CD45^low/−^, CD34^++^CD45^low/−^, and CD34^+++^CD45^low/−^ cell subpopulations with slightly lower intensity of CD34 expression compared to these from the FTPT (Figure 2(d)). The percentage of the cells carrying phenotypes CD34^+++^CD45^low/−^, CD34^++^CD45^low/−^, and CD34^+/low^CD45^low/−^ among the total light-density fraction of FTPT cells was 0.37% (0.16–0.66%, *n* = 6), 0.47% (0.23–0.79%, *n* = 6), and 0.97% (0.36–1.88%, *n* = 6), respectively. In general, the CD34^++^CD45^low/−^ cells from the FTPT were of lymphocyte-like morphology (FSC^low^SSC^low^), whereas the CD34^+^CD45^low/−^ and CD34^+++^CD45^low^ cells were of highly heterogeneous morphology. The multiparameter flow cytometry analysis showed that the CD34^+++^CD45^low/−^ cells carry the immunophenotype CD33^−/low^CD14^−/low^CD235^−^CD19^−^CD7^−/low^CD45RA^−^.

We have also first identified CD34^+/low^CD45^hi^ and CD34^++^CD45^hi^ cells in the FTPT and in the FiTPT in contrast to CB and FL (Figures 2(a)–2(d)). The content of these subpopulations among light-density cells fraction of FTPT was 1.91% (0.50–4.18%, *n* = 6) and 0.38% (0.11–0.79%, *n* = 6), respectively. Their characteristic is a higher expression level of lineage markers compared to the populations described below. The analysis of cell morphology provides evidence that the increase of the CD45 expression on CD34-positive cells coincides with their increase in size and granularity.

**Figure 3:**
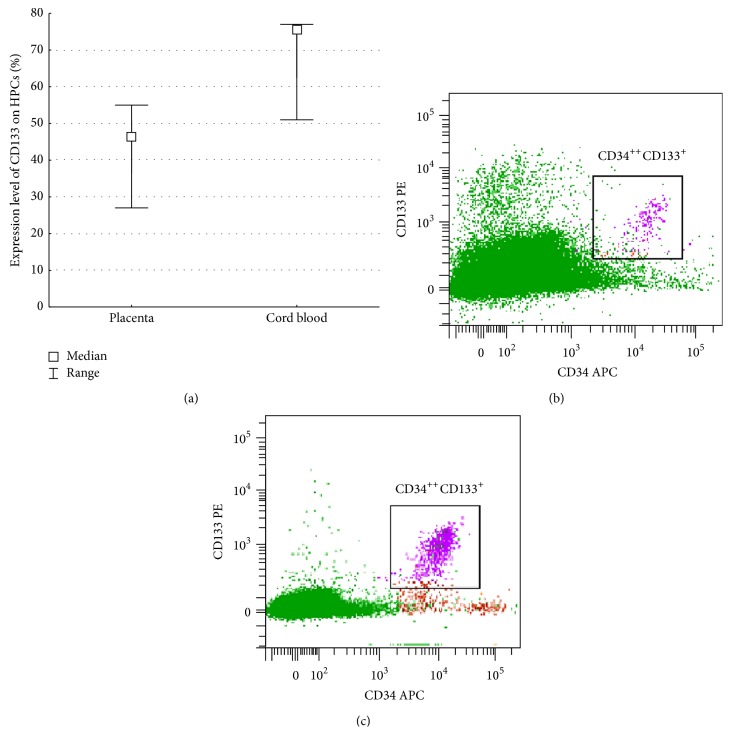
Diagram showing the absence of significant difference in the content of CD133-positive cells among HPCs in FTPT and CB, *n* = 4 (a). The CD133 was expressed by the CD34^++^ cell subset of HPCs in both FTPT (b) and CB samples (c).

**Figure 4:**
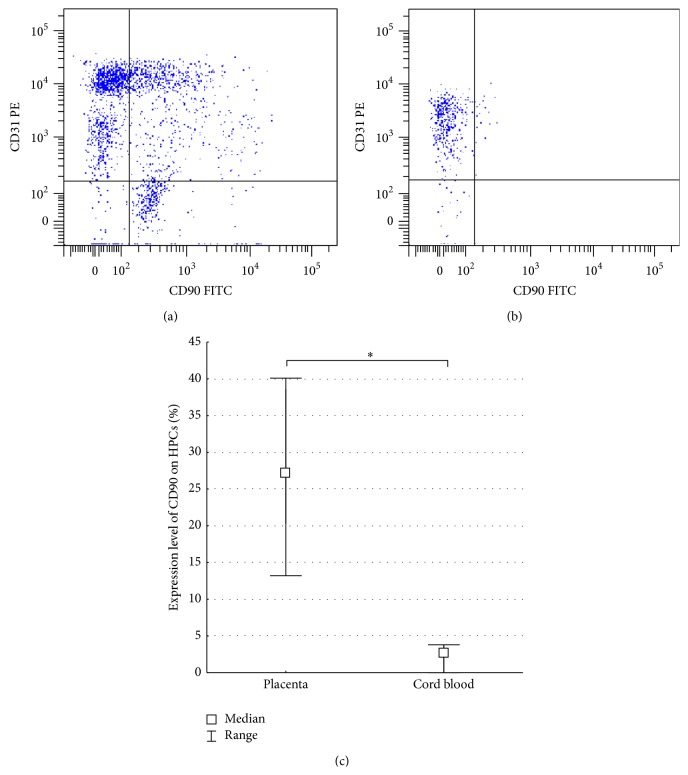
Representative flow cytometry dot plots of CD31 and CD90 expression by HPCs of FTPT (a) and CB origin (b). Diagram showing a significantly higher level of CD90 expression on HPCs from FTPT (*n* = 4) compared to CB, *n* = 5, ^*∗*^ *p* < 0.05 (c).

HPCs from both FTPT and CB expressed the CD133 (Figure 4(a)). The CD133 was shown to be strictly specifically expressed on CD34^++^CD45^low^ subset of both FTPT- and CB-derived cells (see Figures 3(b) and 3(c)).

We have demonstrated that the FTPT-deriving HPCs have a significantly higher CD90 expression level compared to the relevant cells of the CB origin (Figure 4).

The HPCs of the FTPT and CB origin also expressed high levels of CD31 (Figures 4(a) and 4(b)). The HPCs from the FTPT contained two subpopulations with different intensity of the CD31 expression, namely, CD34^+^CD45^low^CD31^hi^ and CD34^+^CD45^low^CD31^low^ cells, whereas the corresponding CB-derived population had only CD34^+^CD45^low^CD31^low^ ones. Furthermore, we observed coexpression of CD90 and CD31 on HPCs (Figures 4(a) and 4(b)).

The samples from the tissues examined (CB, FTPT, and FL) contained immature HPCs with the СD34^+^CD45^low^SSC^low^CD33^−^CD14^−^CD235^−^ phenotype. The content of myeloid/erythroid lineage-committed cells among HPCs of the FTPT was significantly higher than among those of the CB and lower compared to the FL (Table 1).

In addition, the percentage of lymphoid progenitors (СD34^+^CD45^low^SSC^low^CD19^+^, СD34^+^CD45^low^SSC^low^CD7^+^) among all HPCs in FTPT, CB, and FL reach 6.9% (0.9–18.1%, *n* = 6), 3.9% (1.8–6.3%, *n* = 6), and 50.9% (39.6–62.0%, *n* = 7), respectively. Thus, the content of the all lymphoid progenitors in compartment of HPCs was significantly higher in the FL compared to the CB and the FTPT (*p* < 0.05).

**Figure 5:**
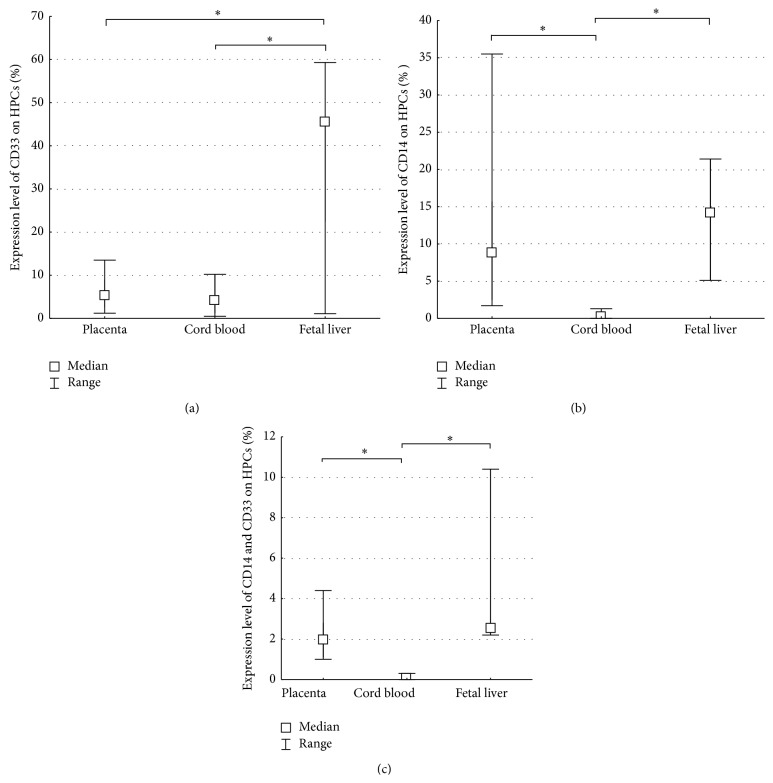
Diagram showing the content of early myeloid progenitors (CD34^+^CD45^low^CD33^+^SSC^low^) among HPCs in the FTPT and the CB was significantly lower than in the FL (a). The FTPT and the FL contained CD34^+^CD45^low^CD14^+^SSC^low^ (b) as well as CD34^+^CD45^low^CD14^+^CD33^+^SSC^low^ (c) late myeloid progenitors in contrast to the CB, ^*∗*^ *p* < 0.05, *n* (placenta) = 6, *n* (CB) = 6, *n* (FL, a) = 3, *n* (FL, b, c) = 7.

The expression level of CD33 on HPCs from the FL was significantly higher than from the CB and the FTPT (Figure 5(a)). Furthermore, CD33^+^ subpopulation of cells was detected in the FiTPT. We have demonstrated that early myeloid precursors are the dominant CB lineage subpopulation of committed hematopoietic progenitor cells.

It should be noted that we were observing a population of late myeloid precursors with the phenotype CD34^+^CD45^low^CD14^+^SSC^low^ in both FTPT and FL in contrast to the CB (Figure 5(b)). Such population was well detectable in the FiTPT. The content of these precursors among HPCs of the FL did not significantly differ from those of the FTPT (Figure 5(b)). In addition, not all CD34^+^CD45^low^CD14^+^SSC^low^ cells were CD33-positive.

CD34^+^CD45^low^CD33^+^CD14^+^SSC^low^ cells were well detectable in the FTPT and in FL but absent in the CB (Figure 5(c)).

**Figure 6:**
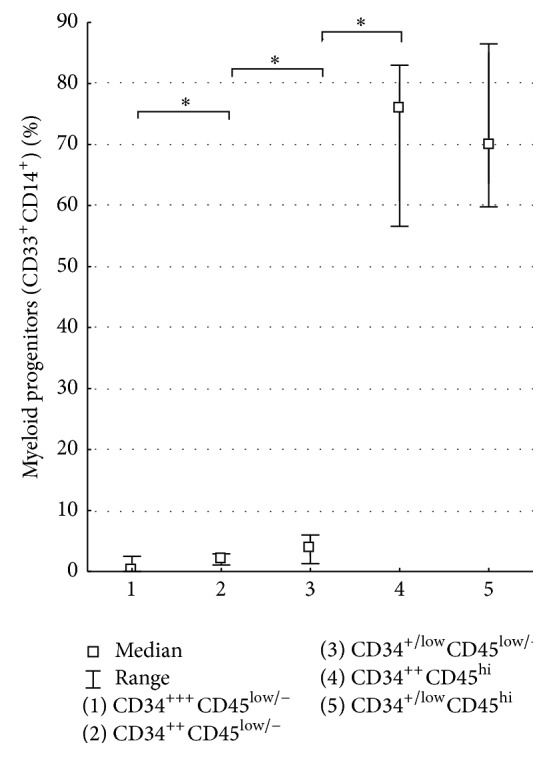
Diagram showing significantly different content of myeloid progenitors expressing CD33 and CD14 among CD34^+++^CD45^low/−^, CD34^++^CD45^low/−^, CD34^+/low^CD45^low/−^, CD34^+/low^CD45^hi^, and CD34^++^CD45^hi^ cells populations in FTPT, ^*∗*^ *p* < 0.05, *n* = 6.

The CD14 expression on the FTPT cells with the immunophenotypes CD34^+++^CD45^low/−^, CD34^++^CD45^low/−^, and CD34^+/low^CD45^low/−^ was 5.2% (2.3–9.4%, *n* = 6), 9.8% (3.5–18.9%, *n* = 4), and 10.9% (4.8–19.1%, *n* = 6), respectively. The expression increase of CD14 was accompanied by the decreasing intensity of CD34 expression on CD45^low/−^ cells of the FTPT origin. Furthermore, the coexpression of CD14 and CD33 was increased on placental CD34-positive cells with growing intensity of CD45 expression and decreasing intensity of CD34 expression (Figure 6).

**Figure 7:**
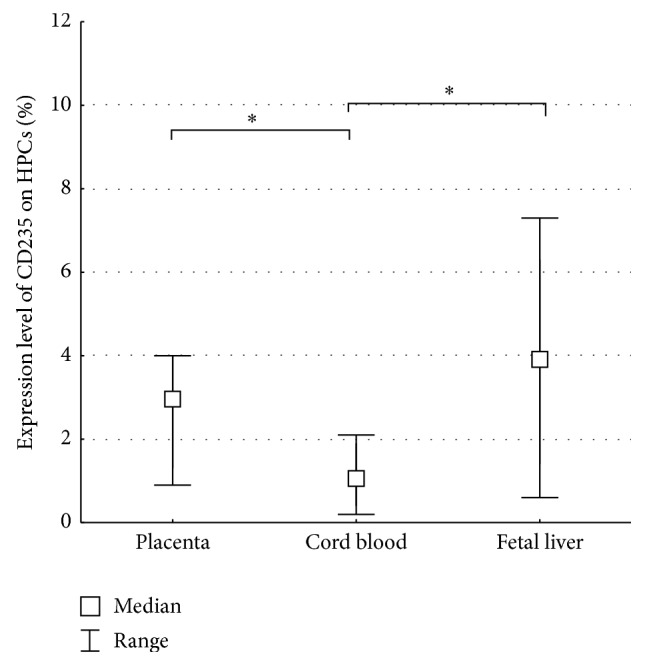
Diagram showing a significantly higher content of erythroid progenitors (CD34^+^CD45^low^CD235^+^SSC^low^) among HPCs from the FTPT and the FL comparing with the CB, ^*∗*^ *p* < 0.05; *n* (placenta) = 6, *n* (CB) = 6, *n* (FL) = 7.

We have demonstrated that the FTPT, similarly to the FL, contains a significantly higher quantity of erythroid progenitors with the CD34^+^CD45^low^CD235^+^SSC^low^ phenotype in compartment of HPCs compared to the CB (Figure 7(a)). We also detected this subpopulation in the FiTPT.

It should be noted that the samples of tissues studied (FTPT, CB, and FL) contained HPCs coexpressing CD235 and CD14 markers. Such cells were also found within erythroid colonies derived from the CB HPCs in semisolid culture medium and they carried CD235^+^CD33^+^CD14^+^, CD235^+^CD33^−^CD14^+^, and CD235^+^CD33^+^CD14^−^ immunophenotypes [15].

**Figure 8:**
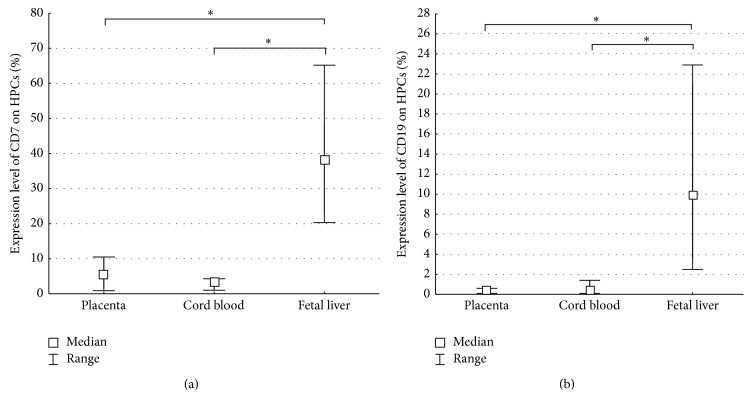
Diagram showing a significantly lower content of T-lymphoid and/or NK-cell progenitors (CD34^+^CD45^low^CD7^+^SSC^low^) among HPCs from the FTPT and the CB compared to the FL (a). Diagram showing a significantly lower content of B-lymphoid progenitors (CD34^+^CD45^low^CD19^+^SSC^low^) among HPCs from the FTPT and the CB compared to the FL, ^*∗*^ *p* < 0.05; *n* (placenta) = 6, *n* (cord blood) = 6, *n* (FL) = 7 (b).

We have found that both the FTPT and the CB contained T-lymphoid progenitors and/or NK-cell progenitors with the CD34^+^CD45^low^CD7^+^SSC^low^ phenotype. The FL contained a significantly higher quantity of T-lymphoid progenitors and/or NK-cell progenitors in compartment of HPCs in comparison with the FTPT and the CB (Figure 8(a)). Among the light-density fraction of FTPT-derived cells we observed 27.1% (14.6–41.8%) of CD7^+^CD45^+^ cells and 10.1% (5.9–15.2%) of CD7^+^CD45RA^+^CD45^+^ ones.

We would like to note that the low level of expression of CD19 was observed on the HPCs from the FTPT and the CB in contrast to significantly higher expression of this marker on FL HPCs (Figure 8(b)).

Similarly to CD14 and CD33, the CD7 and CD19 expression increase was accompanied by the decreasing intensity of CD34 expression on CD45^low/−^ cells from the FTPT and the CB, as we have previously demonstrated [16].

**Figure 9:**
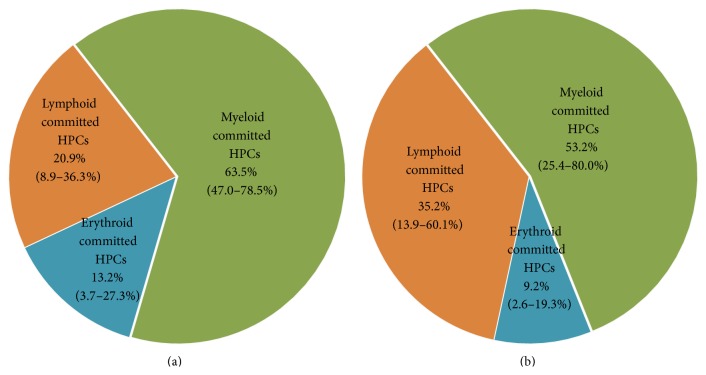
Relative content of myeloid, erythroid, and lymphoid committed HPCs among all committed HPCs analyzed by flow cytometry of light-density cell fraction from FTPT (a) and CB (b) did not differ (*p* < 0.05, *n* = 6).

We compared the ratio of myeloid, erythroid, and lymphoid committed subpopulations among the subset of the committed HPCs from the FTPT and the CB and have not found any significant difference (Figure 9).

**Figure 10:**
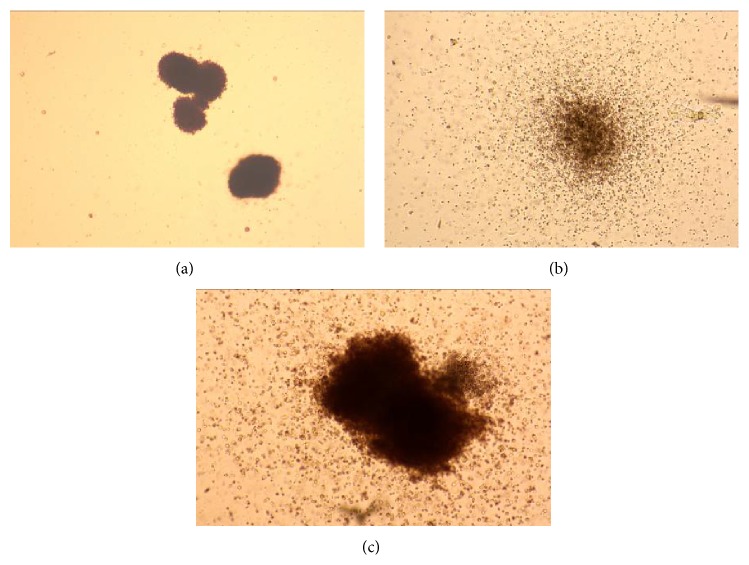
Bright light microscopic appearance of colonies obtained after 14 days of FTPT light-density cell fraction culturing in the MethoCult medium: (а) BFU-E, (b) CFU-GM, and (c) CFU-GEMM (×50).

We have demonstrated that the FTPT contains precursors of hematopoietic cells. On the 14th day of growth they gave rise to all types of morphologically distinguished colonies such as BFU-E (burst-forming unit-erythroid), CFU-E (colony-forming unit-erythroid), CFU-M (colony-forming unit-macrophage), CFU-GM (colony-forming unit-granulocyte, macrophage), and CFU-GEMM (colony-forming unit-granulocyte, erythrocyte, monocyte/macrophage, and megakaryocyte) (Figure 10).

The ratio of the different colony types that were formed by cells from the FTPT and CB did not differ significantly as we have first described previously [17]. The 1 × 10^5^ light-density cells from FTPT contained 112 ± 45 (*n* = 6) colony-forming units. The percentage of BFU-E, CFU-E, CFU-M, CFU-G, CFU-GM, and CFU-GEMM were 41.9% (21.9–56.4%), 6.3% (0.5–17.7%), 7.7% (3.0–14.3%), 6.0% (1.1–14.5%), 19.4% (10.1–30.8%), and 1.0% (0.02–3.4%), respectively.

**Figure 11:**
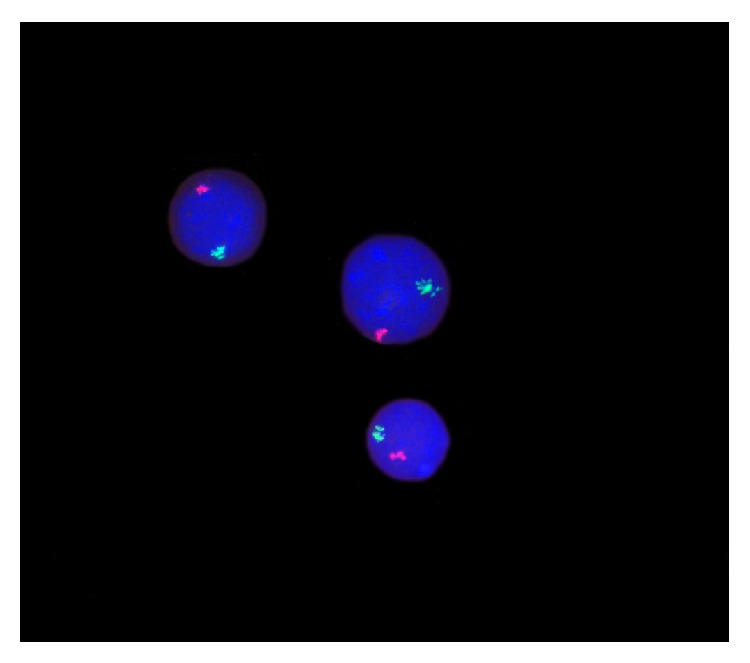
The FISH analysis for X (red) and Y (green) chromosomes indicated the fetal origin of placental hematopoietic cells from different types of colonies generated by progenitor cells from the FTPT in MethoCult medium after 14 days of cultivation (×1,000).

The FISH analysis showed that the nuclei in colony-forming cells from the male FTPT contained X- and Y-chromosomes that permitted to identify the fetal origin of these cells (Figure 11).

**Figure 12:**
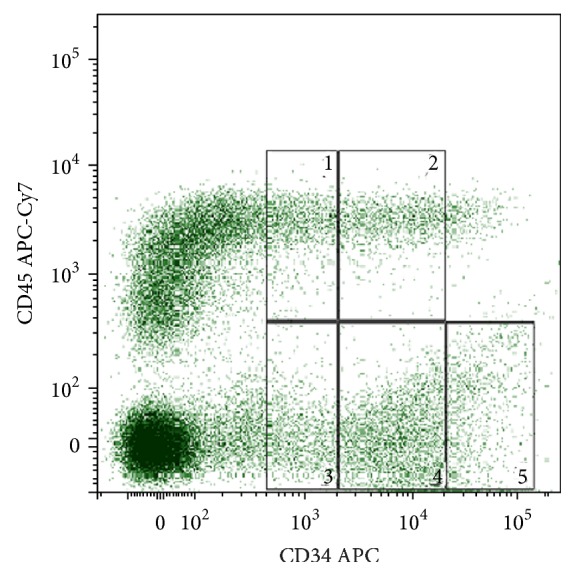
Representative flow cytometry dot plot of CD45 and CD34 expression by light-density fraction of cells from cryopreserved FTPT showing presence of CD34^+/low^CD45^hi^ (1), CD34^++^CD45^hi^ (2), CD34^+/low^CD45^low/−^ (3), CD34^++^CD45^low/−^ (4), and CD34^+++^CD45^low/−^ (5) cells populations.

We propose to carry out the FTPT cryopreservation by a method which we have developed previously and that can save viability of both multipotent mesenchymal stromal cells and the HPCs [12]. We have also analyzed the immunophenotype of the cells from the cryopreserved FTPT in the same way as cells from the native tissue. It was demonstrated that the HPCs from cryopreserved tissue also consist of subpopulations with different levels of intensity of CD34 expression (Figure 12).

**Figure 13:**
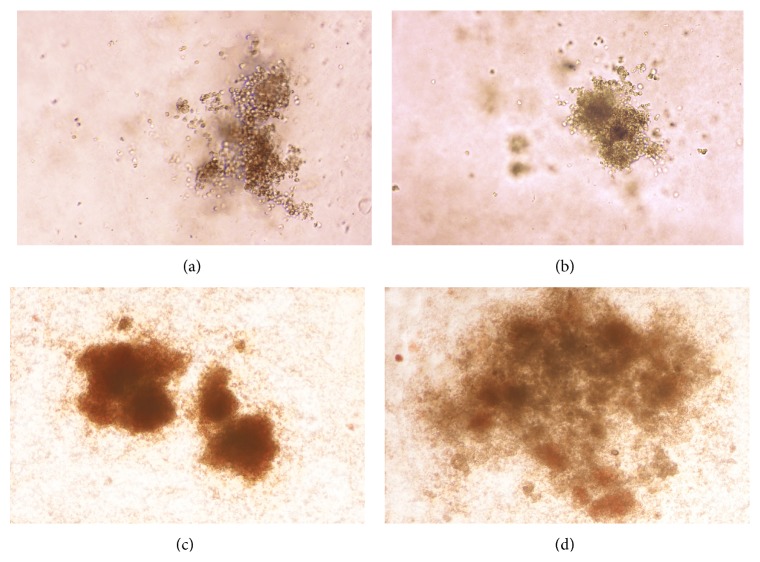
Bright light microscopic appearance of colonies obtained after 21 days of culturing of the cells from cryopreserved FiTPT of 6 weeks of gestation (a, b) and 15 days of culturing of the cells from cryopreserved FTPT (c, d) in MethoCult medium (×100 for a, b and ×50 for c, d).

Using the ISHAGE protocol we have shown that the HPCs content among CD45^+^ cells of the total light-density cell fraction from the cryopreserved and native FTPT was 0.8% (0.1–2.8%, *n* = 3) and 0.4% (0.3–0.6%, *n* = 3), respectively, that does not significantly differ. The content of HPCs among the CD34^+^CD45^low^ cell population was 79.4% (66.2–90.0%, *n* = 3) and the native tissue demonstrates almost the same value as described below. Similarly to HPCs from the native FTPT, the HPCs from the cryopreserved tissue contained cell subpopulations CD34^+^CD45^low^CD31^hi^ and CD34^+^CD45^low^CD31^low^ and expressed CD90. Enzymatically isolated cells fraction from the frozen-thawed tissues (FiTPT and FTPF) also gave rise to different types of colonies (Figure 13).

## 4. Discussion

In our investigation we have first demonstrated similarities and differences of the FTPT HPCs immunophenotypes compared to those of the HPCs from the CB and the FL and their comparative characteristics of colony formation.

The enzymatic treatment causes some changes in the FACS analysis results [14, 18]. Therefore we treated the CB-derived cells with enzymes used for placental cell isolation and then carried out the analysis using the FACS approach.

We suggest that gating by morphology is necessary for correct estimation of the HPCs quantity by the FACS for FTPT and FL because as we first find the percentage of CD34^+^CD45^low^SSC^low^ cells among CD34^+^CD45^low^ ones for these tissues was only near 80%. A low SSC pattern was also useful in the gaiting procedure for the HPCs identification [19, 20]. The CD45 was expressed at low level on almost all precursor cells in the bone marrow [20]. Such level of CD45 was also typical for the HPCs derived from the tissues (CB, FTPT, FiTPT, and FL) studied by us. However, in the FTPT and in the FiTPT we observed cell subpopulations with CD34^+/low^CD45^hi^ and CD34^++^CD45^hi^ immunophenotypes and most of them expressed hematopoietic lineage markers. This fact identifies them as the latest precursors.

For the HPCs isolation from the FTPT, we used two methods. Method #1 allows us to digest the tissue fragments almost completely and to obtain the HPCs for FACS analysis. However, the collagenase reveals a negative effect on cells in dose- and time-dependent manner during the tissue digestion [21]. This finding puts a question of this enzyme's reduced concentration together with its partial replacement by more gentle enzymes to overcome this problem. We have demonstrated that the HPCs content among the viable CD45^+^ cells obtained with collagenase/dispase treatment of the FTPT (method #2) did not differ significantly compared to collagenase/hyaluronidase/DNAase treatment (method #1).

We showed that the expression of lineage markers, namely, CD14, CD7, and CD19, increased simultaneously with the decrease in intensity of CD34 expression on the CD45^+^ cell subset. These data are evidence of the existence of hematopoietic progenitors in the FTPT at different stages of differentiation. These observations are in line with the results of other authors showing that cells of different sources with high level of CD34 expression contained primitive hematopoietic cells; the primitiveness of progenitor cells was closely related to the intensity of CD34 expression [22, 23]. In the adult bone marrow the CD34 antigen density decreased concurrently with the maturation and increase of the CD38 antigen density [24].

The populations of placental cells (FTPT and FiTPT) contained CD34^++^CD45^low/−^, CD34^+^CD45^low/−^, and CD34^+++^CD45^low/−^ cells, which were possibly similar to the cells from the FL; however, these FL populations expressed the CD34 with a slightly lower intensity. In addition, other authors also determine three populations with different levels of intensity of CD34 in human FL [25].

We showed that the HPCs from the CB and FTPT expressed CD133 as it had been also reported in other investigations [8]. But we first noted that the CD133 was expressed strictly specifically on CD34^++^ cells. Strictly speaking, CD133 is a marker of human hematopoietic stem and progenitor cells [26], wherein most of the clonogenic cells were present in the CD34^+^СD133^+^ fraction [27]. This fact evidenced the primitiveness of CD34^++^CD45^low/−^ cell population and is confirmed by our data related to the absence of lineage markers (СD33, CD235, CD19, CD7, and CD45RA) expression. The CD34^++^CD45^low^ cells were reported to express some markers of multipotent primitive hematopoietic progenitors and hematopoietic stem cells; they demonstrated myeloid, erythroid, NK-cells, and B-cells differentiation potential* in vitro* [8]. Therefore, the FTPT and the FiTPT contained primitive HPCs with the CD34^++^CD45^low/−^ phenotype similarly to the FL and the CB.

In our investigation, we first observed that the placental hematopoietic precursors are more heterogenic by their immunophenotype than such cells from the CB and the FL. The fact that we detected progenitors at different stages of differentiation within FTPT and FiTPT suggests that such cells continued to be generated in the placenta and/or they migrated from somewhere to the placental tissue during all periods of gestation until the newborn delivery.

The HPCs from the FTPT expressed CD90 and CD31 markers as was also reported by other researchers [8]. We have first shown that the FTPT contains significantly more CD90-expressing HPCs in comparison with the CB. The CD90 marker is known to be expressed by hematopoietic progenitors with the highest proliferative potential* in vitro* [28–30]. It was also reported that CD34^+^CD90^+^ population contained mainly CD34^+^CD90^+^CD38^low^ cells being early-committed HPCs [31]. Hence, the different level of CD90 expression may evidence the higher content of early-committed HPCs in the FTPT compared to the CB.

The expression of CD31 marker is typical for hematopoietic progenitors [32, 33]. The function of the CD31 of the HPCs was poorly understood, but this protein was found to ensure the interaction with stromal cells of a niche and to be involved into the transendothelial migration [34]. It was also reported the role of CD31 in the differentiation process [35]. Our new finding is that the FTPT tissue contains the CD34^+^CD45^low^CD31^hi^ and CD34^+^CD45^low^CD31^low^ subsets but the CB, however, contains only CD34^+^CD45^low^CD31^low^ subpopulation. This may evidence that circulating HPCs in the CB does not necessarily possess the high intensity of CD31 expression in contrast to the cells residing in the placental tissue and interacting with their niche.

Multiparameter flow cytometry analysis of the expression of lineage markers, such as CD33, CD14, CD235, CD19, CD7, and CD45RA, permitted us first to demonstrate that the population of HPCs from the placental tissues was characterized by a higher content of various lineage-committed progenitors than HPCs from the CB, but a lower content of those cells compared to HPCs of the FL origin.

We investigated the CD33 expression on the HPCs. The CD33 belongs to markers of the early myeloid differentiation stage [36] and the CD34^+^CD33^+^ cells are myeloid progenitors [24], namely, early ones [31]. Similarly to the CB, the FTPT contained early myeloid progenitors. Myeloid progenitor cells that expressed CD33 were the biggest population among all committed CB HPCs that is the fact also reported by other authors [37, 38]. We showed that the FiTPT after 8th weeks of gestation contains the CD34^+^CD45^low^CD33^+^SSC^low^ cells. Other authors also showed a subpopulation of the CD34^++^CD45^low^ and CD34^+^CD45^low^ cells expressing the CD13 and CD33 at second trimester placentas [8]. However, as we show the FL-derived HPCs contained significantly more myeloid progenitors. Studies of the CD13 and CD33 expression show these antigens to be expressed by fetal stem cells in contrast to adult ones [23].

The CD14 is a myeloid marker appearing on monocytes at later stages of maturation that is also expressed on basophils and neutrophils [39, 40] and there are reports that it also has been found on dendritic cells [41] and NK progenitor cells [42]. However, only a few CD34^+^ cells of CB origin coexpressed the CD14 and CD15 [38]. Therefore the expression of CD14 on the HPCs as well as CD33^+^-subset of HPCs from placental tissue and FL may evidence about belonging of all CD14^+^-positive HPCs to the late myeloid progenitors. These cells arose in chorion since 4 weeks of gestation and persist to delivery and they present the first mature leukocyte populations in placenta being the most prevailing [8]. Other authors revealed the presence of macrophage progenitors in the placenta from fetus at the first trimester of gestation even before the formation of fetoplacental circulation. This fact gives evidence that these cells are generated in the placenta* de novo* or to migrate as progenitors through extraembryonic mesoderm [43].

In general, the presence of different subpopulations of myeloid progenitors in the FTPT similar to those found in the FL and their phenotype similarity suggest the existence of placental myelopoiesis.

We first showed that the FTPT as well as FL compartment of HPCs contained higher frequency of the CD235^+^ cells in comparison to CB. The CD235 (glycophorin A) is expressed on mature erythrocytes [44] and primitive embryonic erythroid cells, erythroid and megakaryocytic progenitors, and progenitor cells giving rise to mixed colonies [45]. We observed the CD34^+^CD45^low^CD235^+^SSC^low^ erythroid precursors in the FiTPT at 8 weeks of gestation. It was also demonstrated by other researchers that the placental CD34^+^CD45^low^ cells expressed CD71 and EpoR at 20th weeks of gestation; however, many of these cells did not express the CD235 which may prove their early stage of erythroid differentiation [8]. As it has been reported previously, the final maturation and enucleation of early immature red blood cells occurred in the chorion villi between 5 and 7 weeks of development [43]. Together with the data described above, our results indicated the existence of placental erythropoiesis during the whole period of human fetal development.

We have investigated the expression of CD7 on HPCs and have shown that HPCs of the FTPT, the CB, and the FL contain CD7^+^ cells prevailing in the FL. The CD7 is the earliest marker of T-cell differentiation and is detected in T-cells progenitors [46]. However, these markers are also expressed on the NK-cells at the intermediate stage of differentiation [36], as well as on B-lymphoid progenitors [47] and myeloid progenitors of adult bone marrow and FL [46], but they are found only on a minor subpopulation [48].

We detected in the FTPT at different stages of maturation T-cell populations with phenotypes early T-cells CD7^+^CD45^+^, pro-T-cells CD34^+^CD45^low^CD7^+^SSC^low^, and naive T-cells CD7^+^CD45RA^+^CD45^+^. However, these cell populations might belong to the NK-cells because they expressed both the CD7 [49] and CD45RA [50]. We saw the FTPT compartment of HPCs contained the higher number of the T-lymphoid/NK-cell precursors than the CB, but it did not reach statistical significance that may be due to a relatively small-scope sampling. Some samples of the FTPT contained 10-fold higher levels of the CD34^+^CD45^low^CD7^+^SSC^low^ cells than the CB; it suggests that the T-lymphoid/NK-cell precursors are concentrated in the FTPT extravascular compartment due to their* de novo* generation in placenta tissue or to their migration from placental blood.

The FTPT and the CB compartment of HPCs contained lower levels of B-lymphoid progenitors with the CD34^+^CD45^low^CD19^+^SSC^low^ phenotype than the FL. The CD19 is a marker of B-lymphoid cells which appears very early and does not disappear at later maturation stages [51]. It was earlier found that CD34^++^CD45^low^ cells from human placental tissue in appropriate conditions give rise to B-cells at 19 weeks of gestation [8].

The CFU analysis shows that light-density fraction of FTPT cells gives rise to BFU-E, CFU-E, CFU-M, CFU-GM, and CFU-GEMM colonies of fetal cells that is the fact earlier demonstrated by other researches [9]. It was previously reported that the CFU-Mix colonies generated by CD34^+^ and CD34^−^ cells of the 9th gestation week placenta were of fetal origin [52]. The results of the FISH analysis concerning cultured colonies of FTPT hematopoietic cells show the upper detection limit for fetal origin cells [9].

We observed similar potentials of the FTPT and CB HPCs to multilineage differentiation* in vitro* in spite of a higher content of committed cells in the FTPT compartment of HPCs. However, the ratio of myeloid and erythroid committed HPCs among all committed HPCs did not differ as we first demonstrated by flow cytometry.

Enzyme-isolated cells from cryopreserved FTPT contained hematopoietic progenitors with similar level of expression of CD34 and CD45 compared to ones from native tissue. HPCs derived from cryopreserved and native FTPT had similar properties. The method of cryopreservation of whole placenta by perfusion with cryoprotectants, such as 15% propylene glycol, 14% DMSO, and 14% formamide has been already described [9]. It should be noted that our method of cryopreservation possesses the following advantages: it is technically easy for work and allows us to take a tissue by portions, provides a better penetration of cryoprotectant substances, allows us to use only one cryoprotectant, and does not require high cryoprotectant concentrations that are potentially toxic for cells.

Finally, we assume that the use of CB HPCs together with FTPT HPCs for transplantation will eliminate some disadvantages of the CB transplantation. Isolation of HPCs from FTPT possibly will give opportunity to increase the number of transplanted HPCs. We also suggest that the higher myeloid committing of the FTPT HPCs and the presence of later myeloid progenitors with the CD34^+^CD45^low^СD14^+^SSC^low^ phenotype compared to the CB will give the possibility to overcome a longer recovery time of neutrophil and to accelerate the achievement of their normal level after transplantation. The higher content of early myeloid HPCs in individual leukapheresis products collected from G-CSF-mobilized donors compared to bone marrow and CB grafts provides an earlier engraftment after transplantation [31]. High proliferative potential colony-forming cells are reported to represent a compartment of early myeloid precursors in the FL and the authors suggest the stem cells to be contained by the CD33^+^ cell fraction [53]. A subset of bone marrow CD34^+^ сells contained significantly lower proportions of immature progenitor cells than CB [54] and significantly larger quantities of late-erythroid HPCs and late myeloid HPCs than CB [31]. It might be also important to use for transplantation the HPSCs that reside in their FTPT niches and do not only circulate in the CB.

For our calculations the mean number of total CD34^+^CD45^low^SSC^low^ can be 2.2 × 10^6^ (1.6 × 10^6^–3.0 × 10^6^) cells per placenta if the average normal weight of placenta is 540 g [55], the number of mononuclear cells per 100 g of tissue is 1*∗*10^8^ [56], and the number of СD45^+^ cells is 74% of all mononuclear cells isolated from placental tissue [56].

Therefore, we have shown that fetal hematopoiesis occurs probably in the FTPT and this tissue contains HPSCs at various stages of differentiation that include myeloid, erythroid, and lymphoid lineages commitment. We have developed a method of the placental tissue cryopreservation which preserves the HPSCs viability and offers a new possibility to apply the FTPT as a source of HPSCs for transplantation.

## 5. Conclusions

Our investigation has shown the presence of hematopoietic precursors in the placental tissue at various differentiation stages. The FTPT compartment of HPCs contains a significantly smaller percentage of lineage-committed cells compared to the FL but a significantly larger one compared to the CB. Therefore, the placenta is an attractive source of HPCs for treatment of oncohematological diseases and congenital hematologic disorders not only from the point of view the number of cells but also their characteristics which determine functional properties. The developed method of cryopreservation of FTPT fragments gives opportunity to obtain viable HPCs for clinical application.

## Figures and Tables

**Table 1:** Summaries related to the content differences of committed and uncommitted HPCs among all HPCs in FTPT, CB, and FL.

Source	Cells		
	FTPT	CB	FL
#	1	2	3
Uncommitted cells that were identified by the absence of myeloid and erythroid markers (СD34^+^CD45^low^SSC^low^CD33^−^CD14^−^CD235^−^)	47.8% (29.1–66.9%) *n* = 5	90.3% (78.3–97.8%) *n* = 5	36.2% (21.4–52.5%) *n* = 4
	1-2^*∗*^, 2-3^*∗*^, *p* < 0.05		
Myeloid and erythroid progenitors (СD34^+^CD45^low^SSC^low^CD14^+^, СD34^+^CD45^low^SSC^low^CD33^+^, СD34^+^CD45^low^SSC^low^CD14^+^CD33^+^, and СD34^+^CD45^low^SSC^low^CD235^+^)	19.8% (8.9–33.8%) *n* = 6	7.9% (1.5–18.6%) *n* = 6	52.5% (30.2–74.3%) *n* = 6
	1-2^*∗*^, 2-3^*∗*^, 1–3^*∗*^, *p* < 0.05		

^*∗*^ *p* < 0.05.
